# *GATA4* variant identified by whole‐exome sequencing in a Japanese family with atrial septal defect: Implications for male sex development

**DOI:** 10.1002/ccr3.1851

**Published:** 2018-10-11

**Authors:** Daisuke Shimizu, Satoru Iwashima, Keisuke Sato, Satoshi Hayano, Maki Fukami, Hirotomo Saitsu, Tsutomu Ogata

**Affiliations:** ^1^ Department of Pediatrics Hamamatsu University School of Medicine Hamamatsu Japan; ^2^ Department of Pediatrics Chutoen General Medical Center Kakegawa Japan; ^3^ Cardiac Intensive Care Unit Shizuoka Children’s Hospital Shizuoka Japan; ^4^ Department of Molecular Endocrinology National Research Institute for Child health and Development Tokyo Japan; ^5^ Department of Biochemistry Hamamatsu University School of Medicine Hamamatsu Japan

**Keywords:** atrial septal defect, *GATA4*, male sex development, whole‐exome sequencing

## Abstract

We identified a heterozygous p.(R284H) variant of *GATA4* in a Japanese family with atrial septal defect, including boys with apparently normal male sex development. The findings, together with the previous data, imply that *GATA4 *variants primarily cause congenital heart disease and rarely result in 46,XY disorder of sex development.

## 1 INTRODUCTION

Atrial septal defect (ASD) is a common congenital heartdisease (CHD) characterized by deficiency of the atrial septal tissue in the region of the fossa ovalis.^1^ There are several types of ASD, and the most frequent type is the ostium secundum ASD (ASD2) that results from an enlarged foramen ovale, inadequate growth of the septum secundum, or excessive absorption of the septum primum.^1^ ASD takes place not only as a sporadic form with no identifiable cause but also as a familial form primarily with an autosomal dominant manner, and the prevalence of familial occurrence is known to be highest for ASD among various CHDs.^2^ This indicates that ASD develops not only as a multifactorial disorder subject to various genetic and environmental factors but also as a single gene disorder.

To date, more than 10 causative genes have been identified for ASD, including *GATA4*.^3^ *GATA4* is an evolutionarily conserved zinc finger transcription factor gene that recognizes the “GATA” motif functioning as an important cis‐element in the promoters of many genes, and plays an essential role in the early development of multiple organs, including the heart, ovary, testis, foregut, liver, and pancreas.^4^ *GATA4 *variants have been identified not only in patients with ASD but also in those with various types of CHDs including tetralogy of Fallot (TOF), ventricular septal defect, and atrioventricular septal defect.^5^, ^6^ Furthermore, consistent with the wide expression pattern including the testis, *GATA4* variants have also been found in patients with 46,XY disorder of sex development (DSD) due to impaired testis formation with or without CHD.^6^, ^7^ These findings imply that *GATA4* variants lead to the development of various types of CHD and 46,XY DSD with variable expressivity and reduced penetrance.

Here, we report a *GATA4* variant identified in a family with dominantly inherited ASD2, and clinical and endocrine findings on sex development in two boys with the variant.

## 2 CASE PRESENTATION

**Figure 1:**
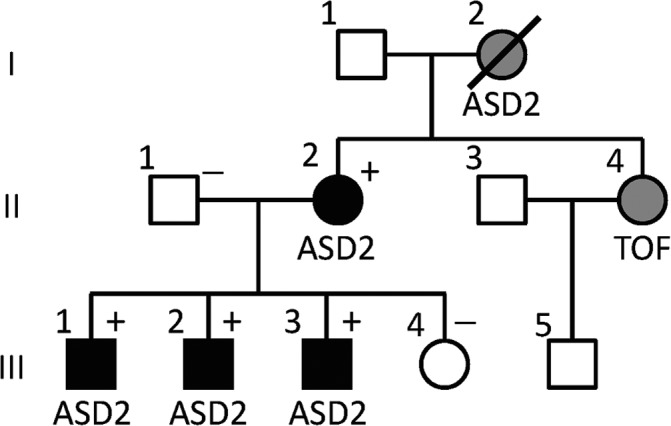
The pedigree of a Japanese family examined in this study. The black, gray, and white symbols indicate subjects with clinically confirmed ASD2, those with allegedly suggested but not clinically confirmed ASD2 or TOF, and those without cardiac disease, respectively. The (+) and (–) symbols indicate molecularly confirmed *GATA4* variant‐positive and *GATA4* variant‐negative subjects, respectively

The pedigree of this Japanese family is shown in Figure 1. The proband (III‐1) was born at 39 weeks of gestation to the mother (II‐2) who was diagnosed as having ASD2 in her childhood and received intracardiac repair (ICR). Allegedly, the maternal grandmother (I‐2) also had ASD2, and the maternal younger sister (II‐4) had TOF. Because of such family history, cardiac assessment was performed for III‐1 at one month of age. Chest X‐ray delineated mildly enlarged pulmonary vessels, and electrocardiography (ECG) revealed incomplete right bundle branch block (iRBBB). Thus, ultrasound cardiography (UCG) was carried out, showing ASD2 and right atrial and ventricular enlargement with moderate pulmonary stenosis (PS). He underwent ICR and pulmonary valvuloplasty at two 7/12 years of age. III‐2 and III‐3 were also found to have similar chest X‐ray findings and iRBBB, and were diagnosed to have ASD2 with right atrial and ventricular enlargement and moderate PS by UCG shortly after birth. III‐2 and III‐3 also received ICR and pulmonary valvuloplasty at five 0/12 and four 3/12 years of age, respectively. Subsequently, although III‐4 was suspected to have patent foramen ovale by fetal UCG at 34 weeks of gestation, she was shown to have no abnormal findings by chest X‐ray, ECG, and UCG.

Except for the CHDs, there was no clinically discernible abnormal finding in the ASD2‐positive III‐1, III‐2, and III‐3. They were born at term with normal birth sizes, and exhibited normal growth and mental development. In the yearly follow‐up observations, physical examinations showed no minor anomalies, and routine blood and urine laboratory tests including hepatic and renal function markers indicated normal findings. On the last examinations at 7, 5, and 3 years of age, respectively, they remained healthy without medication, and bone survey and visceral ultrasonographic studies revealed no abnormal findings. The parents and the ASD2‐negative III‐4 were also clinically normal.

We performed whole‐exome sequencing (WES) with SureSelect Human All Exon V6 (Agilent Technologies, Santa Clara, CA, USA), using leukocyte genomic DNA of II‐1, II‐2, III‐2, III‐3, and III‐4. This study was approved by the Institutional Review Board Committee at Hamamatsu University School of Medicine, and performed after obtaining written informed consent. Captured libraries were sequenced by NextSeq 500 (Illumina, San Diego, CA, USA) with 150‐bp paired‐end reads. Reads were aligned to the reference genome (Human GRCh37/hg19), using BWA‐MEM (version 0.7.12) with default parameters. Duplicated reads were removed by Picard (version 2.9.2), and local realignment and base quality recalibration were performed by GATK version 3.7. Variants were identified with the GATK HaplotypeCaller, and variants with minor allele frequencies of <0.005 in all the following public databases and in‐house database were selected as rare variants: whole genome and WES data for East Asian population in Genome Aggregation Database,^8^ Human Genetic Variation Database,^9^ and allele frequency data of 2049 Japanese individuals.^10^ Final variants were annotated with Annovar.^11^

**Figure 2:**
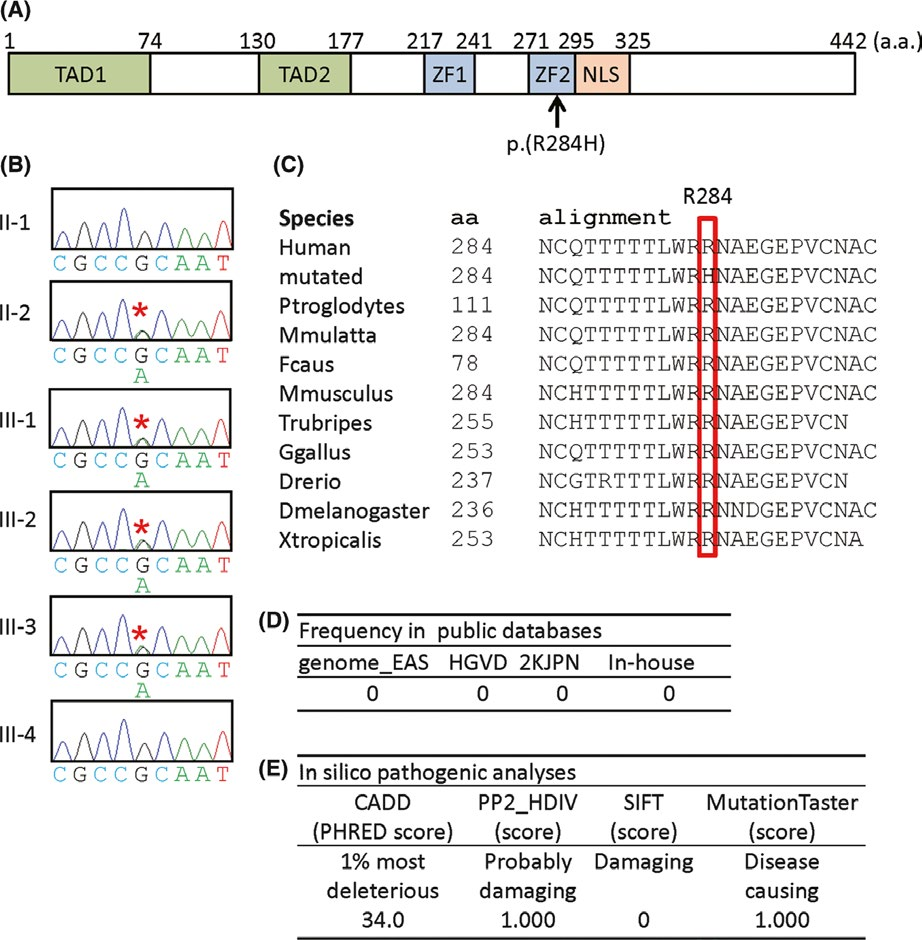
Summary of molecular and in silico analyses. A, Structure of the GATA4 protein and the position of the p.(R284H) variant. The GATA4 protein consists of 442 amino acids and harbors two transcription activation domains (TAD1 and TAD2), two zinc finger domains (ZF1 and ZF2), and a single nuclear localization signal (NLS) The p.(R284H) variant resides on ZF2. B, Electrochromatograms showing the c.851G>A substitution on exon 4 in II‐2, III‐1, III‐2, and III‐3 (indicated with red asterisks). The primers used are as follows: forward, 5'‐CGTGATTCCTCACTCTCTGC‐3'; and reverse, 5'‐TCCAAATGAACAGCCAATTTT‐3'. C, Conservation of the R284 residue among different species. D, Complete absence of p.(R284H) in the public and in‐house databases. The URLs utilized in this study are shown in the footnotes of Table S1. E, In silico pathogenic analyses for p.(R284H). The URLs utilized in this study are shown in the footnotes of Table S1

Consequently, 34 rare variants including nine variants completely absent from the public and in‐house databases were found to be present in the ASD2‐positive subjects (II‐2, III‐2, and III‐3) and absent from the ASD2‐negative subjects (II‐1 and III‐4) (Table S1). We next carried out in silico pathogenicity prediction using four different methods, OMIM survey for diseases caused by mutations of the corresponding genes, and UCSC search for tissue expression pattern of the corresponding gene, thereby identifying a heterozygous missense substitution at exon 4 of *GATA4* (GenBank, NM_002052.4:c.851G>A, p.(R284H); CRCh37, Chr8:11607687) as the most likely candidate variant for ASD2 in this family (Figure 2A, Table S1). This variant was also found in the ASD2‐positive subject III‐1, as well as in II‐2, III‐2, and III‐3 (Figure 2B). The R284 residue was highly conserved (Figure 2C), and this variant was completely absent from the public and in‐house databases (Figure 2D) and was assessed to have high pathogenicity by all the four in silico pathogenic predictions (Figure 2E). Furthermore, this variant was found to be reported as the causative variant in a French family with ASD2.^12^ No other candidate variant for CHD was identified.

After identification of the *GATA4* variant, we investigated the status of male sex development in two variant‐positive subjects (III‐1 and III‐2) (Table 1). Physical examination showed normal male external genitalia with intrascrotal testes of ~2 mL. Chromosome analysis showed a 46,XY karyotype. Gonadotropin‐releasing hormone (GnRH) tests revealed normal basal and GnRH‐stimulated serum luteinizing hormone (LH) values and mildly increased basal and GnRH‐stimulated serum follicle‐stimulating hormone (FSH) values in both subjects. Human chorionic gonadotropin stimulation tests indicated sufficient serum testosterone productions. Serum anti‐Müllerian hormone (AMH) values were obviously low in both subjects.

**Table 1:** Clinical and endocrine findings in male subjects III‐1 and III‐2 with a *GATA4* variant

	III‐1	III‐2
Age at examination, y	7	5
Clinical findings		
External genitalia	Normal	Normal
Other findings	None	None
Serum hormone values		
LH (mIU/mL)	0.4 (0.2‐1.9) → 4.7 (1.1‐6.0)^a^	0.2 (0.2‐1.9) → 5.3 (1.1‐6.0)^a^
FSH (mIU/mL)	3.3 (<0.3‐2.4) → 8.2 (1.9‐7.6)^a^	3.2 (<0.3‐2.4) → 18.5 (1.9‐7.6)^a^
Testosterone (ng/dL)	5.3 (3‐13) → 226.2 (>200)^b^	<5 (3‐13) → 454.8 (>200)^b^
AMH (ng/mL)	16.3 (43.3‐79.3)	17.3 (43.3‐79.3)

AMH: anti‐Müllerian hormone; ASD: atrial septal defect; CHD: congenital heart disease; FSH: follicle‐stimulating hormone; LH: luteinizing hormone.

The values in parentheses represent the age‐ and sex‐matched normal range.^17^, ^18^

a Peak value during a GnRH stimulation test (GnRH 100 μg/m^2^ bolus iv; blood sampling at 0, 30, 60, 90, and 120 min).

b Basal and stimulated values in an hCG test (3,000 IU/m^2^ per dose [max. 5,000 IU] im for three consecutive days; blood sampling on days 1 and 4).

## 3 DISCUSSION

We identified a rare*GATA4 *variant co‐segregating with ASD2 in this Japanese family. In this regard, the absence of this variant in the public and in‐house databases, high pathogenicity predicted by the four in silico analyses, and gene information by OMIM and UCSC survey (Table S1), in conjunction with the previous description of the same variant in a French family with ASD2,^12^ indicate that this variant is responsible for the development of ASD2 in this Japanese family. According to the ACMG Standards and Guidelines,^13^ this variant is regarded as a “likely pathogenic variant,” because this variant is positive for PS1 (same amino acid change as an established pathogenic variant), PM2 (absent from controls), PP1 (co‐segregation with disease phenotype in multiple affected family members), and PP3 (multiple lines of computational evidence in support of a deleterious effect).

ASD2 with PS was the salient cardiac phenotype in this family, although the maternal sister (II‐4) allegedly had TOF. Previous studies have also revealed various*GATA4* variants in familial forms of ASD2 with and without PS.^5^, ^12^ Thus, it is likely that *GATA4* variants frequently cause ASD with and without PS and occasionally lead to other types of CHDs, depending on the effects of other multiple genetic and environmental factors.

No clinically recognizable extra‐cardiac feature was identified in the ASD2‐positive subjects in this family, although it might be possible that some extra‐cardiac feature(s) have remained undetected. This would primarily be consistent with the*GATA4* variant in this family, because previous studies have shown that *GATA4* variants, though they are occasionally accompanied by extra‐cardiac features such as 46,XY DSD^6^, ^7^ and congenital diaphragmatic hernia,^14^ usually lead to isolated CHDs (primarily ASD2). However, lack of extra‐cardiac features is not a common finding in CHDs in general. Rather, CHDs are often associated with extra‐cardiac features, such as brain, skeletal, respiratory, gastrointestinal, and/or genitourinary malformations.^15^, ^16^ In this regard, further studies will serve to clarify underlying causes (eg, single gene variants, oligogenic variants, and multifactorial factors, with variable expressivity and reduced penetrance) of CHDs with and without extra‐cardiac features. Furthermore, such clarification based on both clinical and genetic studies will permit better clinical management and genetic counseling in CHD‐positive patients and their relatives.

Clinical and endocrine studies were performed for male sex development in two variant‐positive boys. In this regard, although well‐masculinized external genitalia and normal LH and testosterone productions indicate well‐preserved Leydig cell function, slightly increased FSH values and obviously low AMH values may suggest more or less compromised Sertoli cell function in the two boys.^17^, ^18^, ^19^ In particular, serum AMH is s a reliable marker of Sertoli cell function.^18^ However, since GATA4 binds to the *AMH* promoter and enhances *AMH* expression synergically with NR5A1,^6^ the *GATA4* p.(R284H) variant may have primarily impaired *AMH* expression, with no clinically discernible deleterious effect on other Sertoli cell function including Sertoli‐germ cell interaction. Indeed, male subjects with the same p.(R284H) variant in the French family are fertile, although they have ASD2.^12^ Thus, the deleterious effect of *GATA4* p.(R284H) variant on testicular function would remain at a subclinical level, if any. It should be pointed out, however, that *GATA4* variants have been identified in five patients with 46,XY DSD.^6^, ^7^ Thus, it is likely that *GATA4* variants lead to 46,XY DSD in rather exceptional subjects, depending on the residual activity of the *GATA4* variants and the predisposing genetic and environmental factors of the variant‐positive subjects.

## 4 CONCLUSION

We identified a *GATA4* variant in a family with dominantly inherited ASD2 by WES. The results, together with the previous findings, imply that *GATA4 *variants primarily lead to congenital heart disease and rarely result in extra‐cardiac features including 46,XY DSD.

## CONFLICT OF INTEREST

The authors declare no conflict of interest.

## AUTHOR CONTRIBUTION

DS: performed whole‐exome sequencing and drafted the manuscript. SI, KS, and SH: performed the patient care and collected clinical information. MF: performed the whole‐exome sequencing in co‐operation with DS. HS: supervised the whole‐exome sequencing and performed the data mining. TO: coordinated the study and wrote the manuscript.

## References

[1] Geva T , Martins JD , Wald RM . Atrial septal defects. Lancet. 2014;383:1921‐1932. 24725467 10.1016/S0140-6736(13)62145-5

[2] Caputo S , Capozzi G , Russo MG , et al. Familial recurrence of congenital heart disease in patients with ostium secundum atrial septal defect. Eur Heart J. 2005;26:2179‐2184. 15980033 10.1093/eurheartj/ehi378

[3] Andersen TA , Troelsen KL , Larsen LA . Of mice and men molecular genetics of congenital heart disease. Cell Mol Life Sci. 2014;71:1327‐1352. 23934094 10.1007/s00018-013-1430-1 PMC3958813

[4] Chen SR , Tang JX , Cheng JM , et al. Loss of Gata4 in Sertoli cells impairs the spermatogonial stem cell niche and causes germ cell exhaustion by attenuating chemokine signaling. Oncotarget. 2015;6:37012‐37027. 26473289 10.18632/oncotarget.6115 PMC4741912

[5] Yang YQ , Wang J , Liu XY , Chen XZ , Zhang W , Wang XZ . Mutation spectrum of GATA4 associated with congenital atrial septal defects. Arch Med Sci. 2013;9:976‐983. 24482639 10.5114/aoms.2013.39788 PMC3902718

[6] Lourenço D , Brauner R , Rybczynska M , Nihoul‐Fékété C , McElreavey K , Bashamboo A . Loss‐of‐function mutation in GATA4 causes anomalies of human testicular development. Proc Natl Acad Sci U S A. 2011;108:1597‐1602. 21220346 10.1073/pnas.1010257108 PMC3029689

[7] Eggers S , Sadedin S , van den Bergen JA , et al. Disorders of sex development insights from targeted gene sequencing of a large international patient cohort. Genome Biol. 2016;17:243. 27899157 10.1186/s13059-016-1105-y PMC5126855

[8] Lek M , Karczewski KJ , Minikel EV , et al. Analysis of protein‐coding genetic variation in 60,706 humans. Nature. 2016;536:285‐291. 27535533 10.1038/nature19057 PMC5018207

[9] Higasa K , Miyake N , Yoshimura J , et al. Human genetic variation database, a reference database of genetic variations in the Japanese population. J Hum Genet. 2016;61:547‐553. 26911352 10.1038/jhg.2016.12 PMC4931044

[10] Yamaguchi‐Kabata Y , Yasuda J , Tanabe O , et al. Evaluation of reported pathogenic variants and their frequencies in a Japanese population based on a whole‐genome reference panel of 2049 individuals. J Hum Genet. 2018;63:213‐230. 29192238 10.1038/s10038-017-0347-1

[11] Wang K , Li M , Hakonarso H . ANNOVAR: functional annotation of genetic variants from next‐generation sequencing data. Nucleic Acids Res. 2010;38:e164. 20601685 10.1093/nar/gkq603 PMC2938201

[12] El Malti R , Liu H , Doray B , et al. A systematic variant screening in familial cases of congenital heart defects demonstrates the usefulness of molecular genetics in this field. Eur J Hum Genet. 2016;24:228‐236. 26014430 10.1038/ejhg.2015.105 PMC4717196

[13] Richards S , Aziz N , Bale S , et al. Standards and guidelines for the interpretation of sequence variants: a joint consensus recommendation of the American College of Medical Genetics and Genomics and the Association for Molecular Pathology. Genet Med. 2015;17:405‐424. 25741868 10.1038/gim.2015.30 PMC4544753

[14] Yu L , Wynn J , Cheung YH , et al. Variants in GATA4 are a rare cause of familial and sporadic congenital diaphragmatic hernia. Hum Genet. 2013;132:285‐292. 23138528 10.1007/s00439-012-1249-0 PMC3570587

[15] Tennstedt C , Chaoui R , Körner H , Dietel M . Spectrum of congenital heart defects and extracardiac malformations associated with chromosomal abnormalities: results of a seven year necropsy study. Heart. 1999;82:34‐39. 10377306 10.1136/hrt.82.1.34 PMC1729082

[16] Egbe A , Uppu S , Lee S , Ho D , Srivastava S . Prevalence of associated extracardiac malformations in the congenital heart disease population. Pediatr Cardiol. 2014;35:1239‐1245. 24823885 10.1007/s00246-014-0922-6

[17] Hasegawa Y (ed.). Normal values in an hCG stimulation test Let’s Enjoy Pediatric Endocrinology (3rd ed.). Tokyo: Shindan to Chiryou‐sha; 1999:260‐262 (In Japanese).

[18] Rey RA , Belville C , Nihoul‐Fékété C , et al. Evaluation of gonadal function in 107 intersex patients by means of serum antimüllerian hormone measurement. J Clin Endocrinol Metab. 1999;84:627‐631. 10022428 10.1210/jcem.84.2.5507

[19] Musnier A , León K , et al. mRNA‐selective translation induced by FSH in primary Sertoli cells. Mol Endocrinol. 2012;26:669‐680. 22383463 10.1210/me.2011-1267 PMC5417138

