# Supplementary material for: GATA4 variant identified by whole‐exome sequencing in a Japanese family with atrial septal defect: Implications for male sex development
Source: Clin Case Rep. 2018 Oct 11;6(11):2229–33. doi: 10.1002/ccr3.1851 (PMC6230668; doi:10.1002/ccr3.1851)
Supplement: Supplementary file 1 [file CCR3-6-2229-s001.pdf]

Supplementary Table S1. Rare variants present in three affected subjects (II-2, III-2, and III-3) and absent from unaffected subjects (II-1 and III-4).

Identified variant		Frequency in public database (MAF)				*In silico* pathogenic analyses				Gene information		
Gene<br>(Location)	Variant<br>(GenGank; GRCh37)	gnomeAD<br>exome_EAS	HGVD	2KJPN	In-house	CADD<br>PHRED score	PP2_HDIV<br>score	SIFT<br>score	MutationTaster<br>score	Phenotype<br>(OMIM)	Inheritance	High expression<br>(UCSC)
**<Variant completely absent from the public and in-house databases>**												
STOX2<br>(Chr. 4q35.1)	NM_020225.2:c.1205C>T<br>p.(P402L)<br>(Chr4:184931196)	0	0	0	0	Deleterious<br>15	Probably<br>damaging<br>1.0	Damaging<br>0	Disease causing<br>1.0			Brain, Pituitary
STOX2<br>(Chr. 4q35.1)	NM_020225.2:c.2149C>A<br>p.(H717N)<br>(Chr4:184932140)	0	0	0	0	Deleterious<br>13	Benign<br>0.12	Tolerated<br>0.3	Disease causing<br>0.95			Brain, Pituitary
THBS4<br>(Chr. 5q14.1)	NM_003248.5:c.635C>T<br>p.(A212V)<br>(Chr5:79354123)	0	0	0	0	Deleterious<br>14	Benign<br>0.003	Tolerated<br>0.37	Polymorphism<br>1.0			Nerve - Tibial
MGAM2<br>(Chr. 7q34)	NM_001293626.1:c.1105_1106del p.(I369fs)<br>(Chr7:141840620 141840621)	0	0	0	0	–	–	–	–			Small Intestine - Terminal Ileum, Minor Salivary Gland
GATA4<br>(Chr. 8p23.1)	NM_002052.4:c.851G>A<br>p.(R284H)<br>(Chr8:11607687)	0	0	0	0	1% most deleterious<br>34	Probably<br>damaging<br>1.0	Damaging<br>0	Disease causing<br>1.0	Testicular anomalies with or without congenital heart disease (# 615542)<br>Atrial septal defect 2 (#615542)<br>Atrioventricular septal defect 4 (#614430)<br>Tetralogy of Fallot (#187500)<br>Ventricular septal defect 1 (#614429)	AD<br><br>AD<br>AD<br>AD<br>AD	Ovary, Heart - Arterial Appendage, Heart - Left Ventricle, Testis
NPY4R<br>(Chr. 10q11.22)	NM_005972.5:c.620T>C<br>p.(L207P)<br>(Chr10:47087403)	0	0	0	0	–	Possibly<br>damaging<br>0.64	Damaging<br>0.04	–			Colon, Small Intestine - Termial Ileum
PIAS1<br>(Chr. 15q23)	NM_001320687.1:c.1A>G<br>p.(M1V)<br>(Chr15:68348222)	0	0	0	0	Non-deleterious<br>0.0061	Benign<br>0.001	Tolerated<br>1.0	–			Cells - EBV-transformed lymphocytes, Testis
ADAD2<br>(Chr. 16q24.1)	NM_139174.3:c.896G>A<br>p.(R299H)<br>(Chr16:84228747)	0	0	0	0	Non-deleterious<br>1.2	Probably<br>damaging<br>1.0	Tolerated<br>0.55	Polymorphism<br>1.0			Testis
PSG2<br>(Chr. 19q13.31)	NM_031246.3:c.519_527del<br>p.(173_176del)<br>(Chr19: 43579688 43579696)	0	0	0	0	–	–	–	–			Transformed fibroblasts
**<Variant not completely absent from the public and in-house databases>**												
SV2A<br>(Chr. 1q21.2)	NM_014849.4:c.1708C>T<br>p.(R570C)<br>(Chr1:149878379)	0.0002	0	0	0	1% most deleterious<br>34	Probably<br>damaging<br>1.0	Tolerated<br>0.18	Disease causing<br>1.0			Brain
CD1D<br>(Chr. 1q23.1)	NM_001766.3:c.32C>A<br>p.(A11E)<br>(Chr1:158150923)	0	0.0043	0.0024	0	1% most deleterious<br>21	Benign<br>0.005	Damaging<br>0.04	Polymorphism<br>1.0			Spleen, Small intestine
FAM163A<br>(Chr. 1q25.2)	NM_173509.2:c.400C>G<br>p.(P134A)<br>(Chr1:179783220)	0.0014	0.0034	0.0005	0	Non-deleterious<br>0.01	Benign<br>0.001	Tolerated<br>1	Polymorphism<br>0.81			Pituitary, Testis
HYAL1<br>(Chr. 3p21.31)	NM_033159.3:c.847G>C<br>p.(V283L)<br>(Chr3:50339541)	0.0004	0.0018	0.0014	0	1% most deleterious<br>24	Probably<br>damaging<br>0.95	Damaging<br>0	Disease causing<br>1.0	Mucopolysaccharidosis type IX (#601492)	AR	Liver, Spleen
TKT<br>(Chr. 3p21.1)	NM_001135055.2:c.1384G>A<br>p.(A462T)<br>(Chr3:53263034)	0	0.0022	0.002	0	1% most deleterious<br>33	Probably<br>damaging<br>1.0	Damaging<br>0.05	Disease causing<br>1.0	Short stature, developmental delay, and congenital heart defects (#617044)	AR	Whole blood
HHIP<br>(Chr. 4q31.21)	NM_022475.2:c.976G>A<br>p.(V326I)<br>(Chr4:145627827)	0.0007	0	0.0005	0	1% most deleterious<br>21	Possibly<br>damaging<br>0.93	Tolerated<br>0.08	Disease causing<br>1.0			Brain

RAD1<br>(Chr. 5p13.2)	NM_002853.3:c.769A>G<br>p.(I257V)<br>(Chr5:34908950)	0.00040	0.0033	0	0	Deleterious<br>15	Benign<br>0.003	Tolerated<br>0.39	Disease causing<br>01.0			Cells - Transformed fibroblasts
AGGF1<br>(Chr. 5q13.3)	NM_018046.4:c.418A>G<br>p.(K140E)<br>(Chr.5:76331470)	0.0012	0	0	0	Non-deleterious<br>0.001	Benign<br>0.000	Tolerated<br>0.89	Polymorphism<br>1.0			Testis, Cells - EBV-transformed lymphocytes
MTUS1<br>(Chr. 8q22)	NM_001001924.2:c.3319G>A<br>p.(D1107N)<br>(Chr8:17510760)	0.000028	0.0039	0.0034	0	Deleterious<br>18	Possibly damaging<br>0.59	Tolerated<br>0.06	Disease causing<br>0.56			Brain - Cerebellar Hemisphere
PCM1<br>(Chr. 8p.22)	NM_006197.3:c.2644A>G<br>p.(R882G)<br>(Chr8:17820790)	0	0.0043	0.0042	0	1% most deleterious<br>26	Probably damaging<br>1.0	Damaging<br>0.01	Disease causing<br>1.0			Testis, Cells - EBV - transformed lymphocytes
XPO7<br>(Chr. 8q21.3)	NM_015024.4:c.2381A>G<br>p.(N794S)<br>(Chr8:21856301)	0.0007	0	0	0	1% most deleterious<br>21	Benign<br>0.44	Tolerated<br>0.42	Disease causing<br>1.0			Testis
FCN2<br>(Chr. 9q34.3)	NM_004108.2:c.694G>A<br>p.(G232R)<br>(Chr9:137778410)	0	0.0046	0	0	1% most deleterious<br>26	Probably damaging<br>1.0	Damaging<br>0	Disease causing<br>1.0			Liver, Adrenal Gland
PPP1R26<br>(Chr. 9q34.3)	NM_014811.3:c.2398G>A<br>p.(A800T)<br>(Chr9:138378754)	0.0009	0.0034	0.0014	0	Non-deleterious<br>0.058	Benign<br>0.001	Tolerated<br>0.73	Polymorphism<br>1.0			Pituitary, Brain
B4GALNT3<br>(Chr. 12p13.33)	NM_173593.3:c.215A>G<br>p.(N72S)<br>(Chr12:644377)	0.0012	0	0	0	1% most deleterious<br>21	Possibly damaging<br>0.80	Tolerated<br>0.21	Disease causing<br>0.99			Stomach
VWF<br>(Chr. 12p13.31)	NM_000552.4:c.6311C>T<br>p.(T2104I)<br>(Chr12:6103315)	0.0004	0.0012	0.0037	0	1% most deleterious<br>28	Probably damaging<br>1.000	Tolerated<br>0.18	Disease causing<br>1.0	von Willebrand disease, type 1 (#193400)<br>von Willebrand disease, types 2A, 2B, 2M, and 2N (#613554)<br>von Willibrand disease, type 3 (#277480)	AD<br>AR,AD<br>AR	Adipose, Breast, Lung
CHD4<br>(Chr. 12p13.31)	NM_001273.4:c.5361+7G>A<br>(Chr12:6686944)	0	0.0012	0.0009	0	–	–	–	–	Sifrim-Hitz-Weiss syndrome (#617159)	AD	Cells - EBV-transformed lymphocytes
VPS13C<br>(Chr. 15q22.2)	NM_020821.2:c.2319G>T<br>p.(Q773H)<br>(Chr15:62269370)	0	0	0.0005	0	1% most deleterious<br>24	Possibly damaging<br>0.90	Damaging<br>0.01	Disease causing<br>1.0	Parkinson disease 23, autosomal recessive, early onset (#616840)	AR	Pituitary, Cells - EBV-transformed lymphocytes, Nerve
TRIP4<br>(Chr. 15q22.31)	NM_016213.4:c.566G>A<br>p.(R189H)<br>(Chr15:64689965)	0	0.0027	s	0	1% most deleterious<br>32	Probably damaging<br>1.0	Damaging<br>0	Disease causing<br>1.0	Muscular dystrophy, congenital, Davignon-Chauveau type (#617066)<br>Spinal muscular atrophy with congenital bone fractures 1 (#616866)	AR<br>AR	Thyroid
SPHK1<br>(Chr. 17q25.1)	NM_182965.2:c.1025A>G<br>p.(D342G)<br>(Chr17:74383279)	0.0011	0.0013	0.0025	0	1% most deleterious<br>23	Probably damaging<br>1.0	Tolerated<br>0.12	Disease causing<br>1.0			Nerve
CBARP<br>(Chr. 19p13.3)	NM_152769.2:c.1093G>T<br>p.(D365Y)<br>(Chr19:1231161)	0.0002	0.0012	0	0	1% most deleterious<br>26	–	–	–			Brain, Pituitary
AP3D1<br>(Chr. 19p13.3)	NM_003938.7:c.1983C>G<br>p.(D661E)<br>(Chr19:2116622)	0	0.0018	0.0016	0	Deleterious<br>13	Possibly damaging<br>0.82	Tolerated<br>0.16	Disease causing<br>1.0	Hermansky-Pudlak syndrome 10 (#617050)	AR	Pituitary, Testis
ZFR2<br>(Chr. 19p13.3)	NM_015174.1:c.1666C>T<br>p.(P556S)<br>(Chr19:3820254)	0.002	0	0	0	Non-deleterious<br>0.006	Benign<br>0.004	Tolerated<br>0.6	Polymorphism<br>1.0			Pituitary, Testis, Brain
CIC<br>(Chr. 19q13.2)	NM_001304815.1:c.1412G>A<br>p.(R471H)<br>(Chr19:42777347)	0	0	0.0005	0	1% most deleterious<br>26	–	–	Disease causing<br>0.98	Mental retardation, autosomal dominant 45 (#617600)	AD	Brain - Cerebellum, Brain - Cerebellar Hemisphere, Testis

CEACAM20 (Chr. 19q13.31)	NM_001102597.2:c.67G>A p.(V23I) (Chr19:45029263)	0	0.0013	0	0	Non-deleterious 0.31	–	–	–		–
ZSWIM3 (Chr. 20q13.12)	NM_080752.3:c.1913T>A p.(L638Q) (Chr20:44507110)	0.0025	0.0012	0	0.0072	1% most deleterious 26	Probably damaging 1.0	Damaging 0	Disease causing 0.99		Testis, Spleen, Cells - EBV-transformed lymphocytes

Shown are non-synonymous rare variants with minor allele frequencies (MAFs) < 0.005 in gnomAD_exome_EAS, HGVD, 2KJPN, and in-house 139 controls.

These variants have been selected as an autosomal-dominant model.

The URLs utilized are as follows; *in silico* analyses were performed by using the default parameters.

1) GenBank (https://www.ncbi.nlm.nih.gov/genbank/).

2) GRCh37 (http://genome.ucsc.edu/).

3) gnom AD (Genome Aggregation Database): http://gnomad.broadinstitute.org/.

4) HGVD (Human Genetic Variation Database): http://www.hgvd.genome.med.kyoto-u.ac.jp/.

5) 2KJPN (Whole-genome sequences of 2,049 healthy Japanese individuals and construction of the highly accurate Japanese population reference panel): https://ijgvd.megabank.tohoku.ac.jp/.

6) CADD (Combined Annotation–Dependent Depletion): http://cadd.gs.washington.edu/score (Current version: 1.3, GRCh37/hg19); PHRED scores of > 10–20 are regarded as deleterious, and those of > 20 indicates the 1% most deleterious.

7) Polyphen-2 Hum Var: http://genetics.bwh.harvard.edu/pph2/ (Current version: 2.2.2, GRCh37/hg19); HumVar scores were evaluated as 0.000 (most probably benign) to 1.000 (most probably damaging).

8) SIFT (Sorting Intolerant From Tolerant): http://sift.jcvi.org/ (Current version: Aug. 2011; GRCh37/Ensembl 63)); Scores of ≤ 0.05 and those > 0.05 are assessed as damaging and tolerated, respectively.

9) MutationTaster: http://www.mutationtaster.org/ (MutationTaster2, GRCh37/Ensembl 69); Alterations are classified as disease causing or polymorphisms, and the high scores of ~1.00 indicate the high probability of disease-causing variant or polymorphism.

10) OMIM (Online Mendelian inheritance in man): http://omim.org/.

11) UCSC genome browser: https://genome.ucsc.edu/.
